# Hens and Health

**Published:** 1872-07

**Authors:** Thos. K. Beecher


### THE BISTOURY.

VOL. VIII. ELMIRA, N. Y., JULY, 1872. NO. 2.

### Communications.

### HENS AND HEALTH.

THOS. K. BEECHER.

Hens and chickens when they used to own themselves and work for a living, were the jungle fowl out in India. Millions of them are living there still, the far-away cousins of our busy barn-yard people. Hunters in our western States, when they hear crowing and cackling, know that they are coming near a farm house; but hunters in India when they hear the same cries know that they are facing the wilderness. Going half round the globe upsets pretty much everything. It is hard to tell what is a sign of what all the world over; white for weddings among us—for funerals among the Chinese; hats off in Congress—hats on in Parliament; stand up to pray among the Scotch—fall down upon your face among the Mohammedans; the weeds of one country are the petted and potted flowers of another. I have seen our big bull thistle, and a beautiful flower it is too, in European gardens; and our familiar mullen does duty abroad as the American velvet plant. Until a man travels he doesen't know much for certain. And if he travels long enough he won't know anything.

But to return to these jungle fowl and their posterity in my barn-yard. I purposed, planned, and performed great things in their behalf.—Not many months ago I spent more hundreds of dollars than I would like to confess to a self-denying home missionary, in constructing a hen-parlor with convenient approaches.

It proved to be an admirable room; so admirable that it caused some envy even in the domestic department of my house. It was proposed to move the laundry and kitchen to the hen-parlor, so light and cheerful was it, and so easy to keep clean. It reached into the hill-side, and was one-half under ground. The out-of-ground part was of double glass and saw the sun all day long. The floor was hard and smooth as stone. The roosts and hen boxes were made of pine worth thirty dollars a thousand. There was a ventilator in my hen-parlor, which is more than any room in my house has; a spring of water unfailing, and sand, and oyster shells, buckwheat, corn, rye, cracked wheat, screenings, with tid-bits from the butchers. In short, all the appliances of the highest hen civilization were gathered in my hen-parlor.

Possibly there may be a higher degree of luxury vouchsafed to poultry in far-away regions among the bloated aristocrats of Great Britain; but, so far as my knowledge goes, hens never had a better setting out than my hens had.

With what result?

This spring, if I have kept the count correctly, there have been eight broods of chickens hatched, varying in numbers from one to ten.—But singularly enough the mothers have proved most unnatural, and looked to me to provide nurses for their children. This became necessary not alone because of their unwillingness, but also their feebleness and worn-out condition generally.

They were genteel Dorkings. The progenitors of my flock took the prize at our last State Fair. I have never been able to learn just how many dollars were paid for them, but they were of pure and perfect breed. In proof of all which, they cultivated every form of feeble health known among the most approved aristocrats. They languished; they pined; they were sleepless; had disturbed dreams, I judge by the cries heard; and after a sufficient amount of languid promenading in the sunshine inside and outside of their parlor windows, hen after hen has died without seeming cause, but with perfect decorum and resignation.

Suspecting that their delicacy of organization might prove unequal to the task of propagating their kind, I took the precaution of adding to their society robust Brahmas of approved reputation. But as usual the Brahmas caught laziness more effectually than the Dorkings strength. By a sort of compulsory pantomime, we one day indicated to a Bramah hen our wishes as to her duty towards her chickens. We put her in a convenient place and surrounded her with her chickens. They did their part faithfully. They cried to her repeatedly. They reached up their little bills and buried their heads in what feathers they could reach upon her columnar legs. But she wasn't going to squat. She had caught the habits of the upper classes in my hen-parlor; if Dorkings can put their children out to nurse, she wasn't going to attend to her children either. I think I do not exaggerate when I say that in all the population of my barn yard, there has not been a symptom of parental affection for months.

In short, I find myself repeating in behalf of this domesticated and civilized jungle-fowl all the experience which doctors are perfecting in behalf of us who are but civilized and domesticated savages.

As if to twit me with my nonsense, one of my hens got through the park palings and ran off, stole a nest in a wretched, cold, damp place, and came off with a brood of thirteen chickens and raised every one of them, contrary to all the decencies and proprieties of life; while other hens that were watched over and provided with boxes and clean straw and sulphur, and I had almost said with gruel and brown stout, were with difficulty held to their duty in the line of incubation, and when at last their broods struggled into being, they proved sickly and short lived orphans. I pay ten cents a head for every chicken that my children brood and tend and bring up to tail feathers and angle-worm hunts in the garden.

I have done a deal of meditating in that hen-parlor of mine. I begin to distrust civilization and the doctors. Every hen in my parlor would be improved by back-sliding towards her ancestral jungle. And there are few of my neighbors whom I meet saluting them and being saluted with the hospital cry—"how do you do, to-day?" "are you well, to-day?" "how is your family, to-day?" who do not testify by this ghostly politeness, how much we have lost by laying off our savagery and living a life of civilization.

I suspect that the way to health is the little rugged path that leads back to nature. I suspect that my hens will all do better when the time comes that I am not able to furnish them a parlor any longer, and not my hens only—but my children too.

